# CRISPR/Cas9 screening reveals Zfp607b as a novel transcription factor regulating myogenesis

**DOI:** 10.1016/j.gendis.2024.101444

**Published:** 2024-10-30

**Authors:** Siyuan Liu, Wei Wang, Zishuai Wang, Chao Yan, Guohao Han, Weiwei Liu, Yuxing Huang, Wangchang Li, Shengsong Xie, Zhonglin Tang

**Affiliations:** a Kunpeng Institute of Modern Agriculture at Foshan, Agricultural Genomics Institute, Chinese Academy of Agricultural Sciences, Foshan, Guangdong 528225, China; b Shenzhen Branch, Guangdong Laboratory for Lingnan Modern Agriculture, Genome Analysis Laboratory of the Ministry of Agriculture, Agricultural Genomics Institute at Shenzhen, Chinese Academy of Agricultural Sciences, Shenzhen, Guangdong 518000, China; c National Clinical Research Center for Infectious Diseases, Guangdong Key Laboratory for Emerging Infectious Diseases, Shenzhen Third People's Hospital, Southern University of Science and Technology, Shenzhen, Guangdong 518112, China; d Key Laboratory of Agricultural Animal Genetics, Breeding and Reproduction of Ministry of Education & Key Lab of Swine Genetics and Breeding of Ministry of Agriculture and Rural Affairs, Huazhong Agricultural University, Wuhan, Hubei 430070, China

Skeletal muscle formation (myogenesis) is a complex process, and transcription factors (TFs) play an important role in controlling the phases of developmental myogenesis, particularly in myoblast proliferation and differentiation.^1^ However, the functions of numerous TFs in myoblast development and myogenesis remain unclear. To systematically identify TFs regulating myogenesis in skeletal muscle, we performed a genome-scale CRISPR-Cas9 loss-of-function (LOF) screen in mouse myoblast cells to identify TFs whose loss contributes to skeletal muscle proliferation. After screening, we identified 855 TFs closely associated with C2C12 cell proliferation. Functionally, we validated that Zfp607b improved skeletal muscle differentiation and regeneration using RNA interference knockdown *in vitro* and lentiviral injection *in vivo*. For the top fold-change genes in the TF knockout cell library, we explored potential mechanisms using transcriptomics and immunofluorescence. In conclusion, we constructed a genome-wide TF knockout cell library in myoblast and identified a novel TF, Zfp607b, that significantly participated in myogenesis and skeletal muscle regeneration. Our findings contribute to the understanding of the ZFP family involved in myogenesis and regeneration and provide a platform for studying the biological function of TFs in the future. Furthermore, our study offers valuable resources for understanding skeletal muscle development and human muscle-related diseases.

In this study, we successfully established a monoclonal C2C12-Cas9 cell line and identified the CRISPR efficiency using Western blot, indirect immunofluorescence assay, and gene editing sequencing. Cas9 proteins were detected via Western blot (Fig. S1A), and the results of indirect immunofluorescence assay showed Cas9 proteins expressed in the cytoplasm of C2C12-Cas9 monoclonal cells, indicated by green fluorescence (Fig. S1B). To examine the effectiveness of the CRISPR system in C2C12-Cas9 cells, we selected three genes (*MAX*, *Zfp607b*, and *Junb*) and transfected them individually with mus-sg expression plasmids into C2C12-Cas9 cells (Table S1). The target sites of *MAX*, *Zfp607b*, and *Junb* were successfully edited in the C2C12-Cas9 genome, and the indel sequences were validated by Sanger sequencing (Fig. S1C). Following the workflow for constructing a mouse genome-wide TF LOF cell library (Fig. 1A), we designed a sgRNA pool targeting 1578 TFs with a total of 12,470 sgRNAs (including 1611 non-targeting control sgRNAs), averaging 7 sgRNAs per TF with high targeting specificity (Table S2). These sgRNAs were combined with a homology arm for lentiviral CRISPR-Cas9 knockout library recombination (5′-TATCTTGTGGAAAGGACGAAACACCGG-sgRNA target sequence-GTTTAAGAGCTATGCTGG AAACAGCATAGC-3′) (Fig. S2A–C). To validate the quality of the lentiviral library,^2^ we transduced it into C2C12-Cas9 cells (Fig. S3A) and compared sgRNA abundance distributions in the lentiviral plasmid library (Fig. S2D) with those in C2C12-Cas9 cells cultured for 60 h post-infection at a multiplicity of infection of 0.18 (Fig. S3B and Table S2). All sgRNAs were effectively expressed in the lentiviral pool, resulting in a 260-fold coverage for all sgRNAs in the musTF LOF library (Fig. S3C and Table S3). However, the absence of 8 sgRNAs targeting different genes led to 99.4% of sgRNAs being expressed in the cell library (Table S4). Nonetheless, all TFs were successfully targeted for knockout within the musTF LOF library. After six days of continuous proliferation, we harvested sgRNA amplicons from the genomes of cells at five passages and performed next-generation sequencing analysis (Table S3). Among the TFs, 681 showed more than a 100% increase, and 174 showed more than a 50% decrease in the musTF LOF library on the sixth day of proliferation. Notably, we identified a novel candidate, Zfp607b, among the top ten enriched genes (Fig. 1B), in four time-point proliferation selection screenings (Table S3), indicating that Zfp607b might play an important role in myogenesis. Additionally, other top-enriched genes from our screening, such as Pax9 and Junb were reported to have played significant roles in muscle development and regeneration. Pax9 was found to be involved in skeletal muscle development,^3^ and Junb was shown to be essential for muscle mass maintenance.^4^ To further analyze the CRISPR screen results, we examined si-Nfatc3 C2C12 cells and found significant inhibition of proliferation-related gene expression (Fig. S4B), as Nfatc3 was the top down-regulated TF in the mutant cell library (Table S3). These findings supported the reliability of our CRISPR screen.

**Figure 1:**
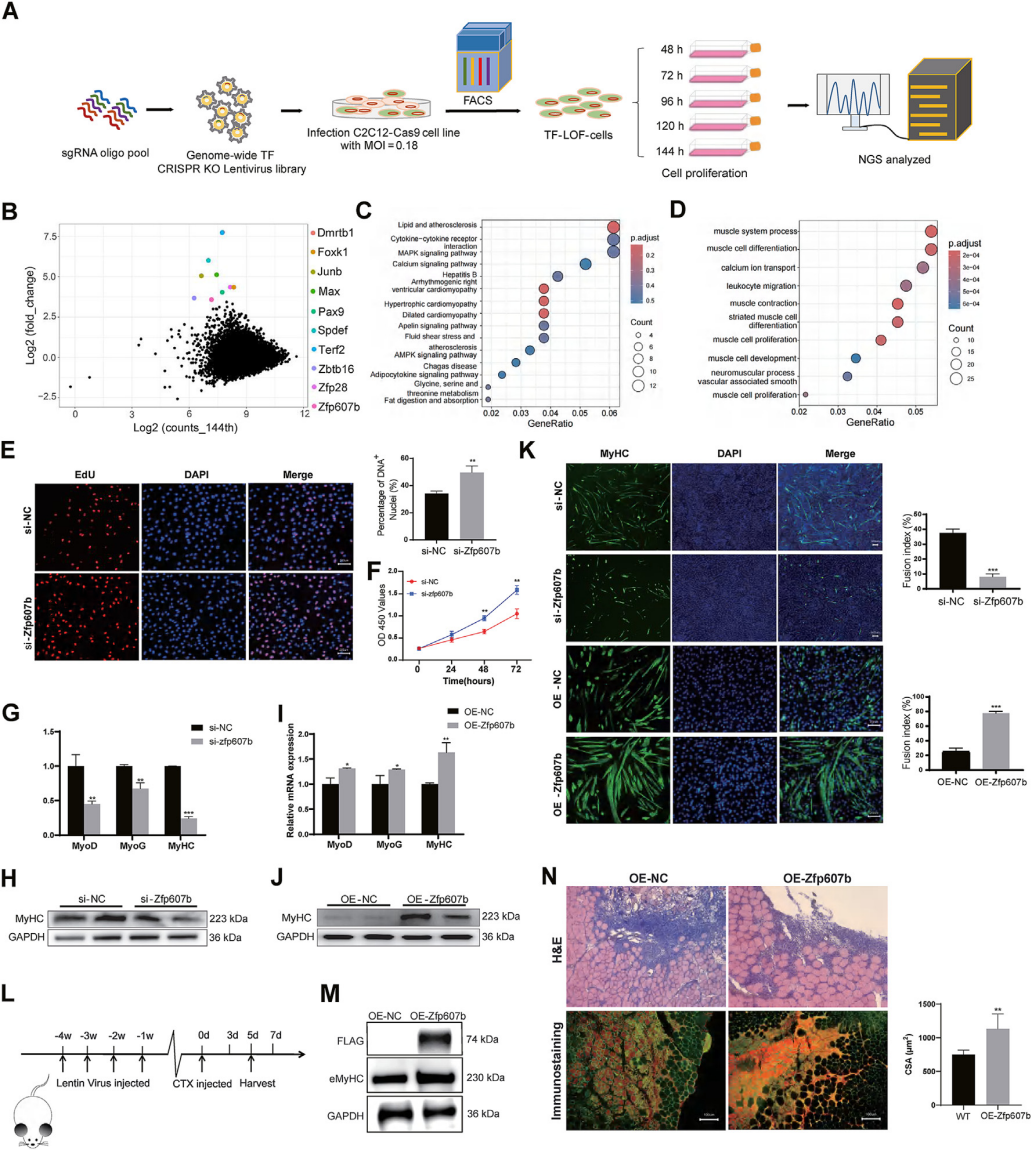
Mouse genome-scale CRISPR transcription factor lost-of-function cell library screened a novel candidate gene Zfp60b, and Zfp607b improved skeletal muscle differentiation and controls critical steps during muscle regeneration. **(A)** Overview of established genome-scale musTF loss-of-function cell library. **(B)** Scatter plots of sgRNAs targeting sequence extent of enrichment in 144 h of mutant cell pool. The top 10 sgRNAs targeting genes are labeled in color. **(C)** Top 15 pathways for the KEGG analysis. *P* < 0.01. **(D)** Top 10 biological processes of the GO analysis (*P* < 0.01). Muscle system process, muscle cell differentiation, and calcium ion transport biological processes were involved in the top 3. **(E)** EdU cell stain analysis in si-Zfp607b C2C12 cells. *n* = 3. Scale bar, 200 μm. **(F)** Myoblasts expressing si-Zfp607b proliferation assay using CCK8. *n* = 3. **(G)** Quantitative reverse-transcription PCR analysis of MyoD, MyoG, and MyHC expression in si-NC and si-Zfp607b mouse myoblasts. **(H)** Western blot (WB) analysis of MyHC and GAPDH expression in si-NC and si-Zfp607b mouse myoblasts. **(I)** Quantitative reverse-transcription PCR analysis of MyoD, MyoG, and MyHC expression in OE-NC and OE-Zfp607b mouse myoblasts. **(J)** WB analysis of MyHC and GAPDH expression in OE-NC and OE-Zfp607b mouse myoblasts. **(K)** MyHC expression in si-Zfp607b and OE-Zfp607b C2C12 identified by indirect immunofluorescence assay and statistics of the fusion index (%). *n* = 3. Scale bar, 200 μm. **(L)** Schematic diagram of lentiviral injection and tibialis anterior muscle injury experiments. *n* = 6. **(M)** WB analysis of Zfp607b, eMyHC, and GAPDH in tibialis anterior (TA) muscles of control and OE-Zfp607b mice at 5 days of cardiotoxin (CTX) injection. **(N)** Hematoxylin and eosin staining and immunostaining analysis of eMyHC fibers in TA muscles of control and OE-Zfp607b mice at 5 days of CTX injection. *n* = 3. Scale bar, 200 μm. CSA, cross-sectional area. Error bars represent standard errors of the mean; *n* = 3; ∗∗∗∗*P* < 0.0001, ∗∗∗*P* < 0.001, ∗∗*P* < 0.01, ∗*P* < 0.05. Nuclei and cytoplasm were colored in blue and pink respectively in hematoxylin and eosin staining. In immunostaining, nuclei and cell membranes were detected by DAPI and FITC, respectively, and eMyHC was colored in red.

Principal component analysis showed that the si-NC cell group was clearly separated from the si-Zfp607b transfected group (Fig. S5A). Transcriptome analysis by RNA sequencing identified 588 differentially expressed genes in si-Zfp607b C2C12 cells, including 230 up-regulated and 358 down-regulated genes (Fig. S5B). Samples from the same condition clustered together, indicating similar library representations in biological replicates. Notably, four main pathways were involved in the si-Zfp607b treatment group: lipid and atherosclerosis, MAPK signaling pathway, calcium signaling pathway, and AMPK signaling pathway (Fig. 1C). Gene Ontology (GO) enrichment analysis predicted 206 biological processes, with the top three being muscle system process, muscle cell differentiation, and calcium ion transport (Fig. 1D). Based on previous studies of gene expression profiles in skeletal muscle during the mouse embryonic and postnatal periods (http://skmatlas.cn/), the expression pattern of Zfp607b was found to be similar to Pax9, Junb, and Spdef (top ten hit genes in Fig. 1B), being up-regulated during the embryonic period and then almost silenced in the postnatal period (Fig. S5D–G). Pax9, Junb, and Spdef have been shown negative correlation with cell proliferation or self-renewal.^3^, ^4^, ^5^ Similarly, the down-regulation of Zfp607b promoted C2C12 cell proliferation (Fig. 1E, F; Fig. S5H) and increased the expression of Ki67, PCNA (proliferating cell nuclear antigen), and cyclin E1 (Fig. S5I, J). Conversely, in OE-Zfp607b C2C12 cells, the expression of Ki67, PCNA, and cyclin E1 (Fig. S6A–C) as well as the number of EdU-positive cells were significantly decreased (Fig. S6D, E), resulting in a decline in the proliferation rate during 72 h of cell culture (Fig. S6F).

As shown in Figure 1G and H, the expression levels of myogenic differentiation factor (MyoD), myogenin (MyoG), and myosin heavy chain (MyHC) were significantly decreased in the si-Zfp607b group, while these markers were notably increased in the OE-Zfp607b C2C12 cells (Fig. 1I, J). The differentiation efficiency of the OE-Zfp607b C2C12 cells was found to be significantly higher compared with the si-Zfp607b cells. The control cells exhibited efficient differentiation, with a fusion index of 38% in si–NC–expressed cells and 25% in OE–NC–expressed cells. In contrast, Zfp607b knockdown lines showed a significantly lower fusion index (8%), whereas OE-Zfp607b-expressed C2C12 cells demonstrated a substantially higher fusion index (78%) (Fig. 1K).

To further validate the regulatory function of Zfp607b in muscle regeneration, 8-week-old mice were injected with a lentiviral control vector or OE-Zfp607b virus once a week for one month. Subsequently, cardiotoxin was injected into the tibialis anterior muscles to induce muscle injury (Fig. 1L; Supplementary Materials and Methods). Five days after injury, mRNA expression levels of Zfp607b were significantly up-regulated in mice injected with OE-Zfp607b virus compared with controls (Fig. S7A), and MyHC expression in OE-Zfp607b mice was up-regulated 15-fold compared with OE-NC mice (Fig. S7B). Additionally, protein levels of Zfp607b and eMyHC were also increased (Fig. 1M). As shown in Figure 1N, hematoxylin and eosin staining of muscle cross-sections at 5 days post-injury revealed faster regeneration in tibialis anterior muscles of mice injected with OE-Zfp607b compared with those injected with OE-NC upon cardiotoxin-induced injury. Further confirmation was provided by immunohistochemical analysis using eMyHC to examine early regenerated myofibers. OE-Zfp607b tibialis anterior muscles showed widespread expression of eMyHC in regenerating fibers, whereas OE-NC muscles exhibited sparser eMyHC expression (Fig. 1N). These results demonstrate that Zfp607b induces skeletal muscle regeneration.

## Ethics declaration

The study was approved by the Ethics Committee of the Institute of Animal Science of CAAS and performed according to the guidelines of the China Biological Studies Animal Care and Use Committee.

## Funding

This work was supported by the National Key Scientific Research Project of China (No. 2023YFF1001100), the 10.13039/501100001809National Natural Science Foundation of China (No. U23A20229), and the Agricultural Science and Technology Innovation Program of China (No. CAAS-ZDRW202406).

## CRediT authorship contribution statement

**Siyuan Liu:** Conceptualization, Writing – original draft, Validation. **Wei Wang:** Validation. **Zishuai Wang:** Visualization. **Chao Yan:** Validation. **Guohao Han:** Validation. **Weiwei Liu:** Validation. **Yuxing Huang:** Validation. **Wangchang Li:** Visualization. **Shengsong Xie:** Software. **Zhonglin Tang:** Writing – review & editing, Funding acquisition.

## Conflict of interests

The authors declared no competing interests.
