# Supplementary material for: CRISPR/Cas9 screening reveals Zfp607b as a novel transcription factor regulating myogenesis
Source: Genes Dis. 2024 Oct 30;12(4):101444. doi: 10.1016/j.gendis.2024.101444 (PMC11981725; doi:10.1016/j.gendis.2024.101444)
Supplement: Multimedia component 1 [file mmc1.docx]

***Supporting information***

**Table of Contents**

Fig. S1. Validate Cas9 expression and effectiveness in C2C12-Cas9 cell line……………2

Fig. S2. Homologous recombination and cell transformed modified………….......………..3

Fig. S3. validated the musTFs LOF library…………..........................................………..4

Fig. S4. qRT-PCR analysis of Ki67, PCNA and CyclinE1 expression in si-Nfatc3 C2C12 cell……………………......………......…………......………….....……......……………..5

Fig. S5.PCA and qRT-PCR analysis in si-Zfp607b and gene profiles analysis in mouse embryonic period and postnatal…………......……………….......……………………..6

Fig. S6.Proliferation detection in the OE-Zfp607b C2C12 cells…………......……………..7

Fig. S7.qRT-PCR analysis of Zfp607b and eMyHC expression in OE-Zfp607b mice……………………......………......…………......………….....……......……………..8


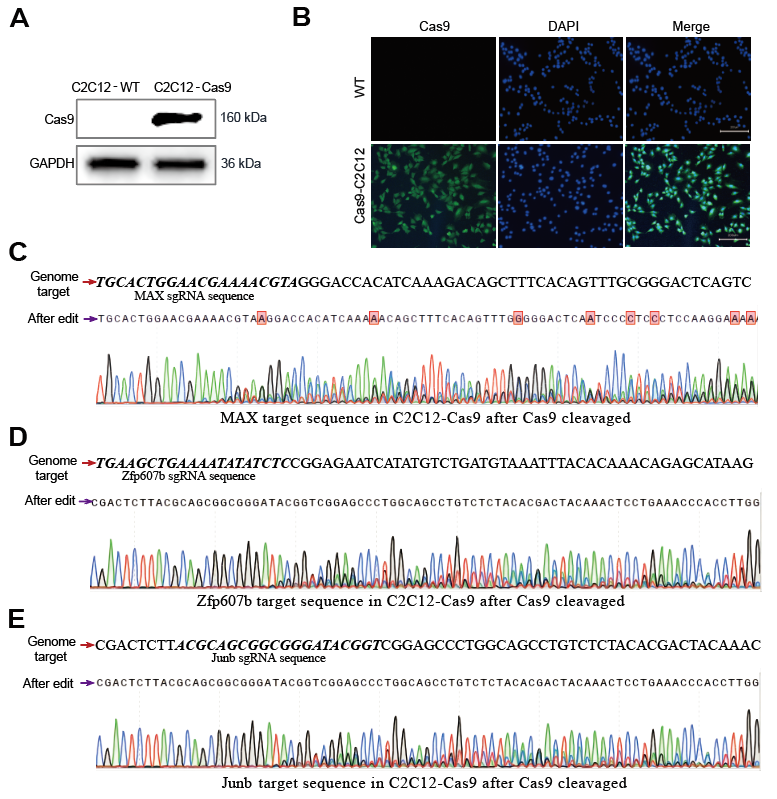


**Figure S1.** Validate Cas9 expression and effectiveness in C2C12-Cas9 cell line. **(A)** Identified Cas9 expression in C2C12-Cas9 cells by WB. **(B)** Evaluation of Cas9 protein expression in C2C12-Cas9 by IFA, scale bar, 200 μm. **(C)** *MAX* genome target sequences editing result in C2C12-Cas9 cells. **(D)** *Zfp607b* genome target sequences editing result in C2C12-Cas9 cells. **(E)** *Junb* genome target sequences editing result in C2C12-Cas9 cells. Cas9: Cas9 proteins were stained in green fluorescence; DAPI, cell nucleus were stained with blue fluorescence; Merge, Cas9 and DAPI overlap images; Genome target, genome sequences of *MAX*, *Zfp607b* and *Junb,* and the location of sgRNAs; After edit, mutation of *MAX*, *Zfp607b* and *Junb* in C2C12-Cas9 after edited and measured by sanger sequencing.


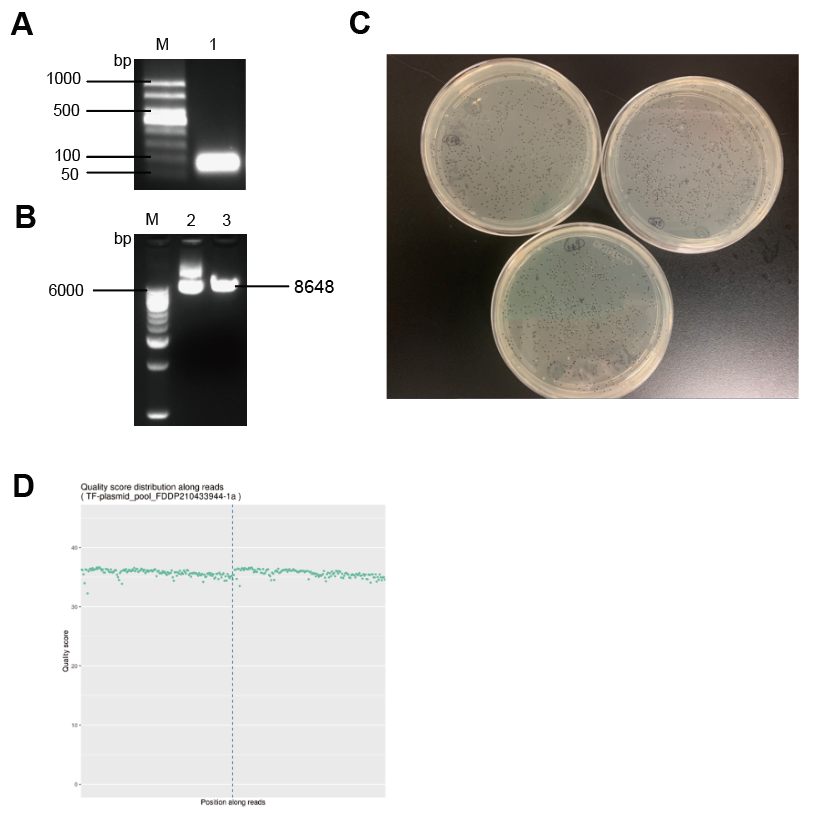


**Figure S2.** Homologous recombination and cell transformed modified. **(A)** sgRNA pool amplification products. **(B)** Lentiviral vector fragments measured by 1.5% agarose gel electrophoresis. **(C)** 10% of the single recombinated system transfected into TStbl3 cells and cultured overnight. **(D)** sgRNA quality score distribution in mus-TF CRISPR KO plasmid library by NGS. M, DNA ladder; 1, sgRNA library amplificons for the mouse genome TFs; 2, recombinated sgRNA-expression vector in circular state; 3, linearized sgRNA-expression vector vector.


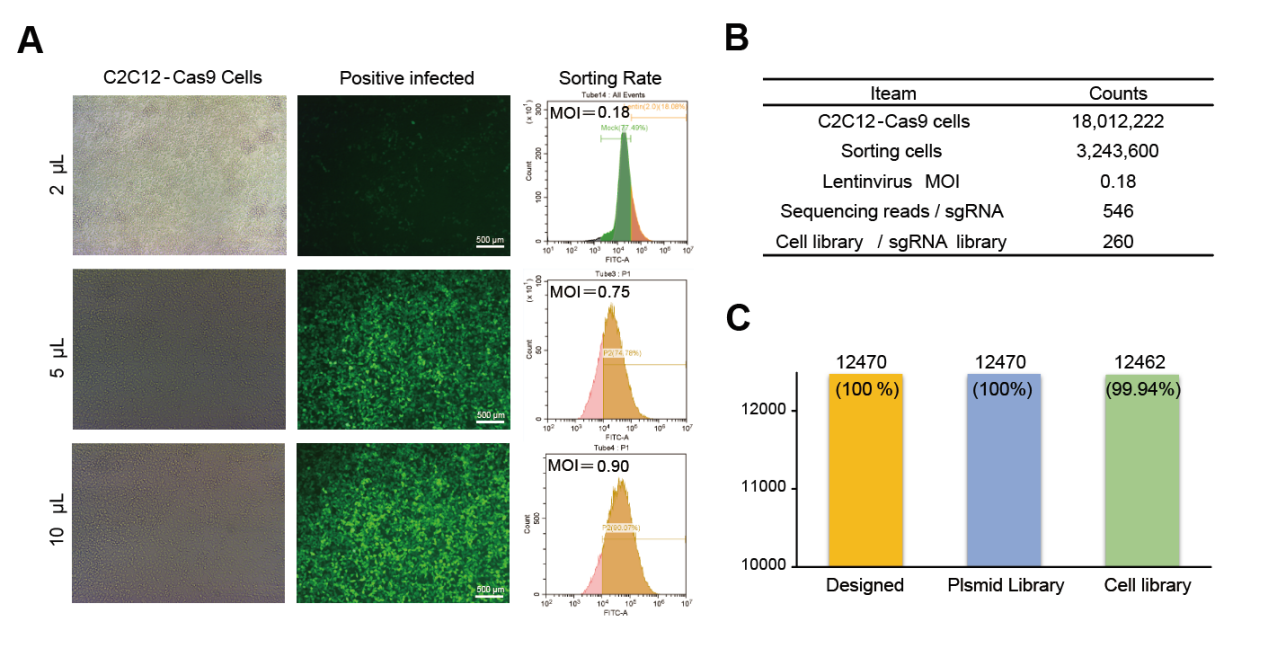


**Figure S3.** validated the musTFs LOF library. **(A)** Infected C2C12-Cas9 cells with sgRNA expressed lentinvirus in different MOI, scale bar, 500 μm (n=3). **(B)** Next generation sequencing (NGS) result of genome-scale knockout TF cell library. **(C)** Table list of sgRNA number in each experiment library. FACS: Fluorescence Activated Cell Sorting. 2 μL, C2C12-Cas9 cells infected with 2 μL sgRNA lentiviral expression library at MOI = 0.18 and the positive infected cell sorting rate is 18.08%; 5 μL, C2C12-Cas9 cells infected with 5 μL sgRNA lentiviral expression library at MOI = 0.75 and the positive infected cell sorting rate is 74.78%; 10 μL, C2C12-Cas9 cells infected 10 μL the sgRNA lentiviral expression library at MOI = 0.90 and the positive infected cell sorting rate is 90.07%.. To assess the quality of the lentiviral library, we amplified the sgRNA constructs in the library and performed , then we found 100% (12,470/12,470) of the initially designed sgRNA sequences were presented in the lentiviral library.


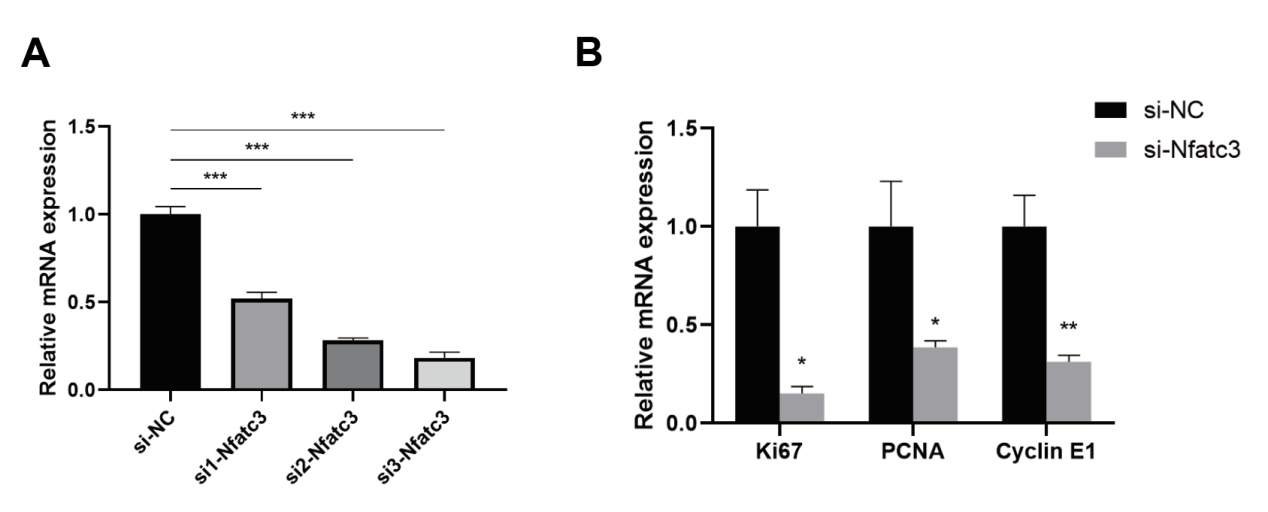


**Figure S4.** qRT-PCR analysis of Ki67, PCNA and CyclinE1 expression in si-Nfatc3 C2C12 cells. **(A)** The efficiency of RNA interference system in si-Nfatc3. **(B)** qRT-PCR analysis of Ki67, PCVA and CyclinE1 expression in mouse myoblasts expressing si-Nfatc3.


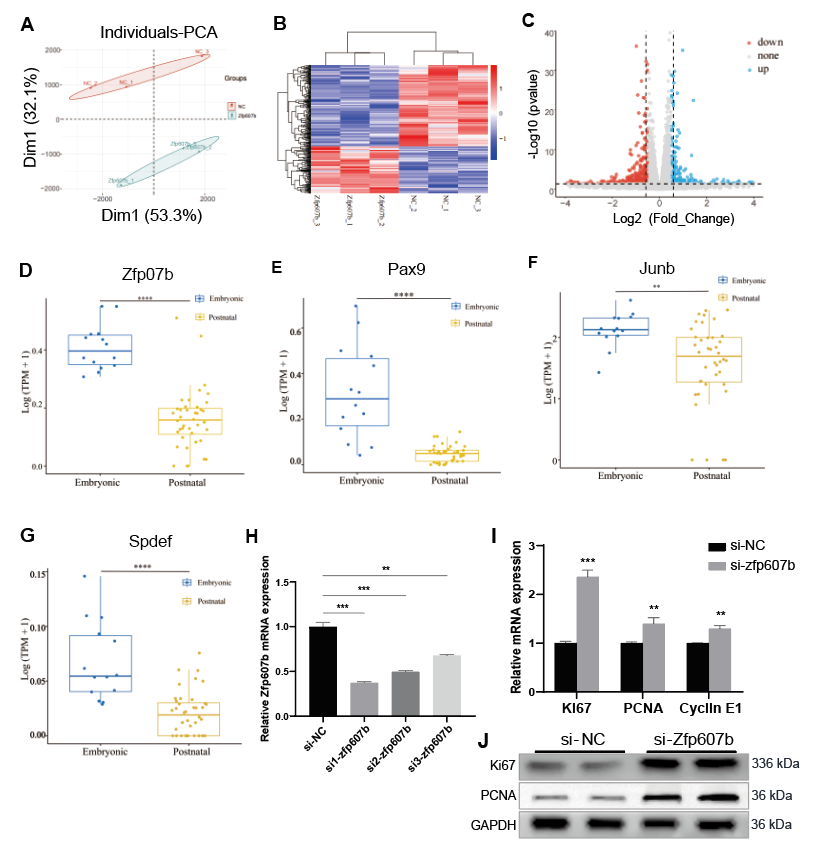


**Figure S5.** PCA and qRT-PCR analysis in si-Zfp607b and gene profiles analysis in mouse embryonic period and postnatal. **(A)** Principal component analysis for si-NC C2C12 cells and si-Zfp607b C2C12 cells. **(B)** Heat map and cluster analysis. **(C)** Volcano plot for richment genes in si-Zfp607b C2C12 cells. up and down represents up-regulated genes and down-regulated genes, respectively. **(D)** Zfp607b expression profiles in skeletal muscle at mouse embryonic period and postnatal. **(E)** Spdef expression profiles in skeletal muscle at mouse embryonic period and postnatal. **(F)** Pax9 expression profiles in skeletal muscle at mouse embryonic period and postnatal. **(G)** Junb expression profiles in skeletal muscle at mouse embryonic period and postnatal. (H) The efficiency of RNA interference system in si-Zfp607b cells. **(I).** qRT-PCR analysis of Ki67, PCVA and CyclinE1 expression in mouse myoblasts expressing si-Zfp607b. **(J)** WB analysis of Ki67, PCVA and CyclinE1 expression in mouse myoblasts expressing si-Zfp607b. Embryonic period include mouse embryonic 15.5 days and embryonic 16.5. Postnatal means 0 day to 5 months mouse. *****P* < 0.0001, ****P* < 0.001, ***P* < 0.01, **P* < 0.05.


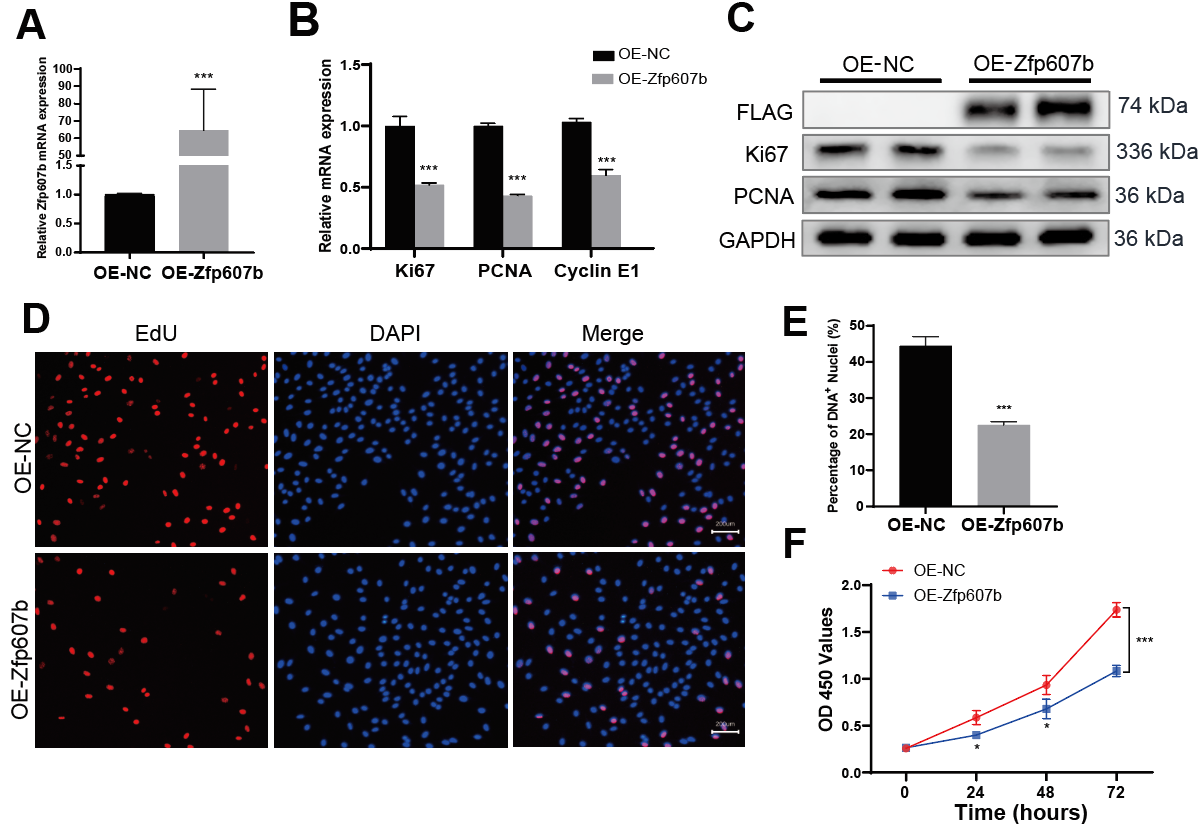


**Figure S6.** Proliferation detection in the OE-Zfp607b C2C12 cells. **(A)** The efficiency of Zfp607b over-experssed system in OE-Zfp607b cells. **(B)** qRT-PCR analysis of Ki67, PCVA and CyclinE1 expression in OE-Zfp607b C2C12 cells. **(C)** WB analysis of Ki67 and PCVA expression in OE-Zfp607b C2C12 cells. **(D)** EdU cell stain analysis in OE-NC and OE-Zfp607b mouse myoblasts. **(E)** PCNA^＋^ cells assay in OE-NC and OE-Zfp607b of D, scale bar, 200 μm (n=3). **(F)** Myoblasts expressing OE-Zfp607b proliferation assay using CCK8. Error bars represent standard errors of the mean (SEM); n = 3, *****P* < 0.0001, ****P* < 0.001, ***P* < 0.01, **P* < 0.05, none means no-significant; EdU-positive cells stained to red; 4’, 6-diamidino-2-phenylindole (DAPI) stained the nuclei to blue-fluorescent.


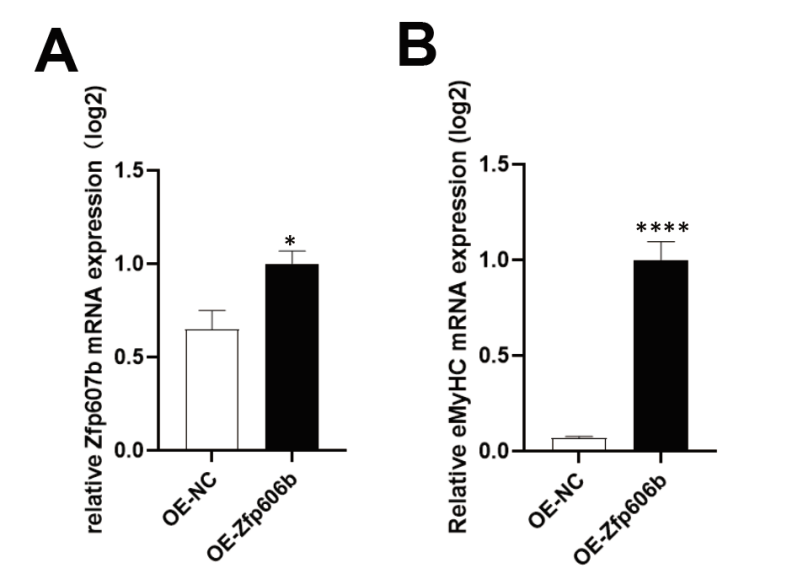


**Figure S7.** qRT-PCR analysis of Zfp607b and eMyHC expression in OE-Zfp607b mice. **(A)** The efficiency of Zfp607b over-experssed system in OE-Zfp607b mice TA. **(B)** qRT-PCR analysis of eMyHC expression in blank control and OE-Zfp607b mice TA.
